# MRNIP interacts with sex body chromatin to support meiotic progression, spermatogenesis, and male fertility in mice

**DOI:** 10.1096/fj.202101168RR

**Published:** 2022-08-03

**Authors:** Samina Kazi, Julio M. Castañeda, Audrey Savolainen, Yiding Xu, Ning Liu, Huanyu Qiao, Ramiro Ramirez‐Solis, Kaori Nozawa, Zhifeng Yu, Martin M. Matzuk, Renata Prunskaite‐Hyyryläinen

**Affiliations:** ^1^ Faculty of Biochemistry and Molecular Medicine University of Oulu Oulu Finland; ^2^ Research Institute for Microbial Diseases Osaka University Suita Japan; ^3^ Department of Comparative Biosciences University of Illinois at Urbana‐Champaign Urbana Illinois USA; ^4^ University of Texas Health Science Center San Antonio Texas USA; ^5^ Department of Pathology & Immunology Baylor College of Medicine Houston Texas USA; ^6^ Center for Drug Discovery Baylor College of Medicine Houston Texas USA

**Keywords:** diplotene, liquid–liquid phase separation, male infertility, pachytene, subnuclear, XY body

## Abstract

Meiosis has a principal role in sexual reproduction to generate haploid gametes in both sexes. During meiosis, the cell nucleus hosts a dynamic environment where some genes are transcriptionally activated, and some are inactivated at the same time. This becomes possible through subnuclear compartmentalization. The sex body, sequestering X and Y chromosomes during male meiosis and creating an environment for the meiotic sex chromosome inactivation (MSCI) is one of the best known and studied subnuclear compartments. Herein, we show that MRNIP forms droplet‐like accumulations that fuse together to create a distinct subnuclear compartment that partially overlaps with the sex body chromatin during diplotene. We demonstrate that *Mrnip* ^*−/−*^ spermatocytes have impaired DNA double‐strand break (DSB) repair, they display reduced sex body formation and defective MSCI. We show that *Mrnip* ^*−/−*^ undergoes critical meiocyte loss at the diplotene stage. Furthermore, we determine that DNA DSBs (induced by SPO11) and synapsis initiation (facilitated by SYCP1) precede *Mrnip* expression in testes. Altogether, our findings indicate that in addition to an emerging role in DNA DSB repair, MRNIP has an essential function in spermatogenesis during meiosis I by forming drop‐like accumulations interacting with the sex body.

**Abbreviations**
ATM: ataxia telangiectasia mutated
ATR: ataxia telangiectasia and Rad3 related
Brca1: breast cancer 1, early onset
CON: control
Ddx3x: DEAD‐Box Helicase 3, X‐linked
Ddx3y: DEAD‐Box Helicase 3, Y‐linked
DMC1: DNA meiotic recombinase 1
DSB: DNA double‐strand break
Fthl17: ferritin heavy polypeptide‐like 17
H1T: testicular H1 histone
H3S10p: Phospho‐Histone H3 phosphorylation at serine 10
HET: heterozygote
Hormad1: HORMA domain‐containing 1
Hormad2: HORMA domain‐containing 2
KAP1: KRAB‐associated protein‐1
KO: knockout
KOMP: knockout mouse project
Mdc1: mediator of DNA damage checkpoint 1
MLH1: MutL homolog 1
MLH3: MutL homolog 3
MRN complex: Mre11 Rad50 Nbs1
MRNIP: Mre11 Rad50 Nbs1 interacting protein
MSCI: meiotic sex chromosome inactivation
NBS1: Nibrin
P: postnatal day
Parp2: poly (ADP‐ribose) polymerase family member2
PCNA: proliferating cell nuclear antigen
Pgk1: phosphoglycerate kinase 1
Pgk2: phosphoglycerate kinase 2
RAD50: DNA repair protein Rad50
RAD51: DNA repair protein Rad51 homolog 1
Rbmy: RNA‐binding motif protein, Y chromosome
RNA POL II: RNA Polymerase II
Rnf212: ring finger protein 212
ssDNA: single stranded DNA
SYCP1: synaptonemal complex protein 1
SYCP3: synaptonemal complex protein 3
Tex11: Testis expressed gene 11
Ube1y1: ubiquitin‐activating enzyme, Y chromosome
Usp26: ubiquitin specific peptidase 26
Utp14a: UTP14A small subunit processome component
Utp14b: UTP14B small subunit processome component
γH2AX: H2A histone family member X phosphorylated at serine 139

## 1 INTRODUCTION

Meiosis is an essential process of sexual reproduction, involving one round of DNA replication and two rounds of chromosome segregation leading to the production of haploid gametes during meiosis I and II.^1^ The process of meiosis is thought to have been present in the last eukaryotic common ancestor and is present in all major eukaryotic lineages.^2^ Meiosis I and II have four stages: prophase, metaphase, anaphase, and telophase, similar to mitosis.^1^ Prophase I is subdivided further into four stages: leptotene, zygotene, pachytene, and diplotene.^3^ Characteristic events during meiosis are the pairing of homologous chromosomes, genetic exchange mediated via double‐strand break (DSB) formation and repair, and the separation of homologous and sister chromatids.^1^, ^3^

Meiotic recombination, the reciprocal exchange of chromosomal DNA between parental chromosomes, is initiated in the leptotene stage of prophase I by the formation of programmed DSBs by topoisomerase II‐like enzyme SPO11.^4^, ^5^ While DNA breaks, occurring spontaneously or in response to exogenous DNA damage, can be lethal for somatic cells, DSBs are required for homologous chromosome pairing and synapsis in most eukaryotic germ cells.^6^, ^7^ DNA DSBs activate complex repair machinery. The covalently bound SPO11 is removed from DNA by the conserved MRN complex comprised of three proteins: MRE11, RAD50, and NBS1.^8^ This leads to the activation of ataxia telangiectasia‐mutated (ATM) and ataxia telangiectasia mutated and Rad3‐related (ATR) kinases signaling to promote DSB repair.^9^, ^10^, ^11^ The formation of ssDNA by resection of the DSB serves as a substrate for RAD51 and DMC1, meiosis‐specific proteins, binding to initiate homologous pairing and strand exchange.^7^, ^12^, ^13^, ^14^

Alignment of the homologous chromosomes begins at the leptotene stage when two pairs of sister chromatids form axial elements through association with a proteinaceous structure called the synaptonemal complex. First, Synaptonemal complex protein 3 (SYCP3) starts to localize on the lateral axial elements of the synaptonemal complex. Later, during the zygotene stage, Synaptonemal complex protein 1 (SYCP1) begins to localize in transverse elements of homologous chromosomes. Formation of the synaptonemal complex is required for homologous recombination to occur in the pachytene stage.^15^, ^16^ Some of the DNA DSBs mature into cross‐overs, occurring at a minimum of one per chromosome pair.^17^ The MutL family proteins MLH1 and MLH3 are directed to these cross‐over sites resulting in the formation of cross‐overs.^18^ The absence of MLH1 affects the maintenance of pairing between the homologous chromosomes.^19^

The occurrence of DSBs leads to one more important event, the phosphorylation of the histone variant H2AX at serine 139 (γH2AX) during the leptotene stage of prophase I.^20^ Once synapsis occurs on autosomes, the γH2AX signal is sequestered onto the unpaired regions of sex chromosomes in early pachytene to form the subnuclear compartment sex body also called XY body. The sex body acquires a characteristic appearance by mid‐pachytene as an ellipsoid, membrane‐less structure encapsulating XY chromosomes in spermatocytes and is characteristically recognized as γH2AX‐positive chromatin staining.^21^, ^22^, ^23^ The sex body is the site where XY bivalents in prophase I undergo meiotic sex chromosome inactivation (MSCI).^21^, ^24^ The MSCI is a part of a larger epigenetic event referred to as meiotic silencing of unsynapsed chromosomes (MSUC) that functions to silence any unsynapsed chromosome regions on autosomes.^21^ Meiotic silencing is triggered by asynapsis. The chromosomes that are unsynapsed as they enter pachytene continue to express DNA DSB markers such as RAD51.^13^ MSCI is essential for male germline formation and its failure leads to the elimination of spermatocytes in the mid‐pachytene stage of meiotic prophase I.^21^, ^25^, ^26^, ^27^ Deletion of genes associated with meiotic sex chromosome inactivation, such as *Brca1*, *H2ax*, *Atr*, *Parp2*, *Mdc1*, and *Hormad2* leads to mis‐expression of lethal sex‐linked genes followed by severe defects in meiotic progression or arrest.^20^, ^28^, ^29^, ^30^, ^31^, ^32^

The MRN complex interacting protein (MRNIP) was discovered as a protein involved in DNA DSB repair in somatic cells through interaction with MRN complex proteins.^33^ It was also reported that MRNIP protein plays a role in maintaining genome stability^33^ and acts as a replication fork protection factor in mitosis.^34^ The most recent study showed that MRNIP forms liquid‐like condensates in the somatic cell nucleus to facilitate DSB end resection and DNA DSB repair.^35^ This demonstrates MRNIP association with liquid–liquid phase separation, a biophysical phenomenon enabling macromolecules to condense and create co‐existing phases.^35^ The report of a *Mrnip* knockout mouse model has shown that male mice were subfertile and states that *Mrnip* is probably responsible for homologous recombination through its interaction with the MRN complex in meiosis.^36^ However, it remains unknown when and in which cells MRNIP is expressed in the testis, the mechanism of how *Mrnip* contributes to the progression through meiosis, and what upstream genetic factors regulate *Mrnip* expression.

In this study, we report that MRNIP is expressed in mid‐pachytene to diplotene spermatocytes and becomes partially associated with sex body chromatin. We show that *Mrnip* deletion leads to compromised DNA DSB repair, reduced sex body formation, and defective MSCI. Additionally, we identified that *Spo11* and *Sycp1* are critical for downstream *Mrnip* expression. Overall, *Mrnip* is essential for meiotic progression, spermatogenesis, and male fertility in mice.

## 2 MATERIALS AND METHODS

### 2.1 Animals and tissues

Mrnip^tm1a(EUCOMM)Wtsi^ (herein referred to as *Mrnip* ^*+/*−^) mice were acquired from the Wellcome Trust Sanger Institute's knockout mouse project and maintained in a mixed C57BL/6J, (C57BL/6N)/129SvEv background. The null allele in these mice was generated through splicing to a *LacZ* trapping element present in the targeting cassette.^37^

*Mrnip* mice were housed in a specific pathogen‐free animal facility in individually ventilated cages under controlled conditions of light (12 h light/12 h dark). Animal handling was conducted in accordance with: the Institutional Animal Care and Use Committees of Baylor College of Medicine, Houston, USA; the Research Institute of Microbial Disease at Osaka University; the Finnish Animal Ethics Committee license (34/2018) and the institutional animal care policies, which fully meet the requirements of European Union Directive 2010/63/EU and the European Convention for the protection of vertebrate animals used for experimental and other scientific purposes (ETS No. 123, appendix A). Genotyping primers are available in Table S1.

*Spo11* and *Sycp1* mice were housed in the Animal Care Facility at the University of Illinois at Urbana‐Champaign, Urbana, IL, USA under 12 h dark/12 h light cycles and at 22 ± 1°C. Animal handling and procedures were approved by the UIUC Institutional Animal Care and Use Committee. The genotyping was done as published earlier.^38^, ^39^ Multiple human tissues were acquired from the Human Tissue Acquisition and Pathology (HTAP) core using BCM IRB‐approved protocol H‐14435 (Baylor College of Medicine, USA). Wild‐type and heterozygous mice were used as controls.

### 2.2 Histology and immunostaining

#### Histology

Testes were fixed in Bouin's Fixative and stained with periodic acid‐Schiff's reagent (PAS, Sigma Aldrich) as described in Ref. [40]. The sections were observed and imaged using DM LB2 (Leica) and Zeiss Axio Imager motorized histology (Zeiss) microscopes.

#### Immunofluorescent staining for cryo‐sections

Adult male testes were fixed in 2% paraformaldehyde (PFA)/PBS for 3 h at 4°C, then washed briefly in PBS, placed in 30% Sucrose/PBS, followed by incubation in a 1:1 mixture of 30% Sucrose/PBS: OCT. Testes were embedded in OCT, frozen on dry ice, and sectioned (10 μm). Control and KO sections were placed on the same slide and stored at −80°C. The primary and secondary antibodies and dilutions are available in Table S2. Nuclei were stained with DAPI.

*Immunofluorescent staining with paraffin‐embedded specimens* was also done as described previously^41^ with some modifications. Briefly, testes were dissected in PBS, fixed in 4% PFA overnight at 4°C, washed briefly in PBS, dehydrated through an ethanol series, embedded in paraffin, and sectioned (5 μm). After paraffin removal and rehydration, antigen retrieval was done by boiling slides in 0.01 M citric acid buffer, pH 6, for 20 min followed by blocking in PBS containing 5% fetal bovine and 5% goat serum for 1 h. The slides were stained with primary and secondary antibodies listed in Table S2 and Hoechst 33342 (Invitrogen). The specimens were mounted with Immu‐Mount (Fisher Scientific) and imaged by confocal microscopy (Olympus Fluoview FV10‐ASW) at Biocenter Oulu Tissue Imaging Center.

### 2.3 TUNEL assay

TUNEL assay for apoptotic cell detection was performed according to the manufacturer's instructions (Roche). Briefly, testes were fixed in 4% PFA, dehydrated through an ethanol series, embedded in paraffin, sectioned at 5 μm, and stained according to the assay instruction. Testes from three mice of each genotype were analyzed. Apoptotic cells were calculated in at least 100 seminiferous tubule cross‐sections and three sections per testis. The result is presented as an average of apoptotic cells per 100 tubules.

### 2.4 Testicular nuclear spreads

Testicular nuclear spreads were prepared as described in Ref. [42] with some modifications. Shortly, the tunica albuginea was removed from adult testes, ground between a pair of serrated tweezers and pipetted up and down (1 ml pipette tip) to release spermatocytes in 1 ml of 1XPBS (pH 7.4). The volume was adjusted to 10 ml with 1XPBS (pH 7.4). Cells were passed through a 40‐μm nylon mesh filter (Falcon) to remove tubules and debris. Cells were pelleted by 7 min centrifugation (100× *g*). The supernatant was removed, and pellet resuspended in 1 ml of hypotonic buffer (17 mM Sodium citrate; 50 mM Sucrose; 30 mM Tris–HCl, pH 8.2–8.4 adjusted with 0.5 M NaOH) supplemented with dithiothreitol (0.5 μM) and incubated for 30 min at RT protected from light. The cell suspension was centrifuged for 7 min (100× *g*), the supernatant was removed, and the cell pellet was gently resuspended in 0.3–1 ml of 100 mM sucrose solution (pH 8.2, set by 0.5 mM Borate buffer). The objective slides (Super Frost Plus (Fisher Scientific)) were dipped in 1% PFA (1% PFA, 0.15% Triton X‐100, pH 9.2, set by 0.5 M NaOH) and 12 μl of cell suspension was applied to the PFA‐coated slide dropwise and then allowed to spread by tilting the slide. The slides were kept in a humidified chamber for 2 h, then air‐dried. Prepared nuclear spreads were stored at −80°C.

Before proceeding to immunostaining, the spreads were air‐dried for 20 min at RT and washed with 1XPBST (PBS, 0.1% Triton X‐100). Blocking was done with 5% fetal bovine and 5% goat serum for 1 h, followed by overnight incubation with primary antibodies, three PBST washes, and 1 h incubation with secondary antibody was performed. The primary and secondary antibodies are listed in Table S2. Nuclei were stained with Hoechst 33342 (Invitrogen). The specimens were mounted with Immu‐Mount (Fisher Scientific) and imaged by confocal microscopy (Olympus Fluoview FV10‐ASW) at Biocenter Oulu Tissue Imaging Center.

### 2.5 Metaphase spreads

Adult mouse testes were harvested from control and knockout mice respectively and treated using a modified protocol published by Evans et al.^43^ Shortly, the tunica albuginea was removed, and testes were washed three times in 2.2% sodium citrate. The tubules were teased apart, pipetted up and down using a plastic Pasteur pipette for about a minute, and incubated for 15 min. The cell suspension was centrifuged at 700× *g* for 5 min at room temperature. The supernatant was discarded and 3 ml of 1% sodium citrate was added and centrifuged as described above.

The cells were incubated overnight at +4°C in 3:1 methanol: glacial acetic acid fixative. Fresh fixative was added to the cells before slide preparation. The slides were prepared using a modified previously published protocol.^44^ First, the Super Frost Plus (Fisher Scientific) slides were presoaked for 15–20 min in the fixative. A cell suspension drop of 25 μl was pipetted on the slides while keeping them on a metal rack over 80°C water bath to steam for a couple of seconds before leaving them to air dry completely. Slides were stained with nuclear dye Hoechst 33342 (Invitrogen) for 20 min followed by three 1XPBS washes, 5 min each, and mounted using Immu‐Mount (Fisher Scientific). Imaging was performed using Zeiss LSM780 (Zeiss) and Zeiss LSM700 (Zeiss) confocal microscopes. A total of 228 and 216 metaphase spreads were quantified from 3 control and 3 *Mrnip* ^*−/−*^ mice, respectively.

### 2.6 RNA isolation, reverse transcription‐ and quantitative real time‐polymerase chain reaction

Mouse cDNA was prepared from multiple adult tissues of C57BL6J/129SvEv hybrid mice and testes. Briefly, tissues were dissected and snap frozen in liquid nitrogen. RNA was extracted using TRIzol (Invitrogen) or RNeasy Protect Mini kit (Qiagen) following the manufacturer's protocol. 0.8–1 μg RNA was transcribed to cDNA using First Strand cDNA Synthesis Kit (Thermo Fisher Scientific) or qSCRIPT cDNA supermix (Quanta) following the manufacturer's protocol. The generated cDNA was used to perform reverse transcription‐ (RT‐PCR) and quantitative real‐ time‐polymerase chain reaction (qRT‐PCR). The primers used are listed in Table S1. QRT‐PCR was performed as described earlier.^45^ Briefly, cDNA was diluted at 1:10 and 1 μl was used for qRT‐PCR in a total volume of 10 μl. The qRT‐PCR program consisted of 40 cycles at 95°C for 30 s and at 60°C for 1 min in a CFX96 Real‐Time System (BioRad) thermocycler. *Gapdh* was used for normalization by the CT method.^46^ A minimum of three mouse testes were used per genotype and qRT‐PCR was performed in duplicates. The real‐time qRT‐PCR results are presented as the average ± SD.

### 2.7 Fertility testing

Sexually mature control (HET*)* and *Mrnip* ^*−/−*^ male mice were housed with wild‐type females for at least 3 months. Copulation was confirmed by checking for vaginal plugs and the number of pups in each cage was recorded.

### 2.8 Sperm motility analysis

Sperm motility was analyzed as described earlier.^40^ Shortly, cauda sperm were extracted from control (HET) and *Mrnip* ^*−/−*^ littermates and incubated for 30 min in human tubular fluid (HTF, Millipore) media supplemented with bovine serum albumin (0.3 mg/ml) at 37°C (capacitating conditions). Sperm samples were diluted, analyzed, and imaged using Hamilton Thorne's CEROSII sperm analysis system (software version 1.5.2; Hamilton Throne Biosciences, Beverly, MA).

### 2.9 Western blot analysis

Testes were dissected and snap frozen in liquid nitrogen. Tissues were solubilized in T‐per (Thermo Fisher) lysis buffer supplemented with protease and phosphatase inhibitors (cOmplete, EDTA‐free, Roche) and PhosSTOP (Roche) or in 1XTBS with 1% Triton X‐100 and protease inhibitors (Pierce). We used Bis‐tris 4%–12% pre‐cast gels (Invitrogen) or 12.5% hand‐casted polyacrylamide gels to load 20 μg of total protein. Proteins were transferred to nitrocellulose membranes. Blocking was done in 5% BSA for phospho antibodies (Table S2) and in 5% milk for others for 1 h at room temperature. Primary antibodies (Table S2) were diluted in 5% BSA buffer and incubated overnight at 4°C followed by TBST (1XTBS and 0.1% Tween‐20) washes. Appropriate secondary antibodies (Table S2) were diluted 1:5000 and incubated for 1 h at room temperature. Bands were visualized by Western Bright ECL (Advansta) or West Femto ECL substrate (Fisher Scientific) using a ChemiDoc™ XRS imager (BioRad).

### 2.10 Statistical analysis

Statistical analysis was performed using a two‐tailed Student's *t*‐test (*p ≤ .05, **p ≤ .01, and ***p ≤ .001) by Microsoft Excel and GraphPad Prism 9.3.1 (GraphPad, San Diego, CA, USA). Data represents the mean ± standard deviation (±SD). At least three mice of each genotype were used in each experiment unless indicated differently.

## 3 RESULTS

### 3.1 *Mrnip* expression is enriched in mouse and human testes

**FIGURE 1:**
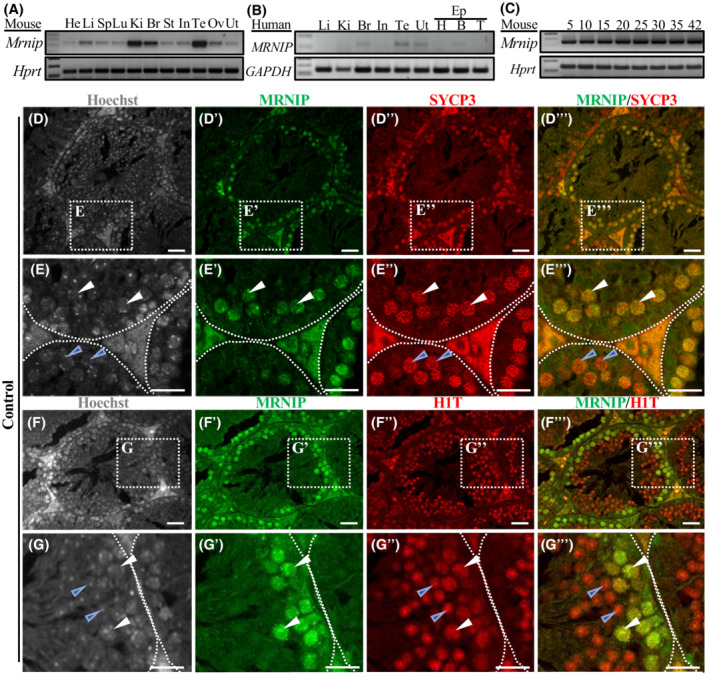
(A) Mouse multi‐tissue RT‐PCR profile of *Mrnip* gene expression shows that *Mrnip* is ubiquitously expressed and enriched in the kidney, brain, and testes. *Hprt* gene was used as a control. (B) Human multi‐tissue RT‐PCR analysis indicates that *MRNIP* expression is enriched in the brain, testes, and uterus. *GAPDH* gene was used as a control. (C) *Mrnip* gene is expressed in mouse testes by P5 and onwards as indicated by RT‐PCR analysis. *Hprt* gene was used as a control. (D–E‴) Immunostaining showing MRNIP expression in adult testes in relation to SYCP3 staining. (E–E‴) Inserts of larger magnification. (D, E) Hoechst staining depicting nuclei. (D′, E′) MRNIP (green) and (D″, E″) SYCP3 (red) antibody staining shows that staining colocalizes in pachytene spermatocytes (D‴, E‴, white arrows) but not in earlier stages (D‴, E‴, blue arrows). (F–G‴) Immunostaining showing MRNIP expression in testes in relation to H1T staining. (G–G‴) Inserts of larger magnification. (F–G) Hoechst staining depicting nuclei. (F′, G′) MRNIP (green) and (F″, G″) H1T (red) antibody staining shows colocalization in spermatocytes from mid‐pachytene to diplotene (F‴, G‴, white arrows) but not in round spermatids (F‴, G‴ blue arrows). Heart (He), liver (Li), spleen (Sp), lung (Lu), kidney (Ki), brain (Br), stomach (St), intestine (In), testis (Te), ovary (ov), uterus (Ut), epididymis (Ep): head (H), body (B), and tail (T). Scale bar (D–G‴) 20 μm.

The mouse *Mrnip* gene has seven exons and is located on chromosome 11. Our RT‐PCR analysis demonstrates that *Mrnip* is ubiquitously expressed in multiple mouse tissues with the strongest expression in the kidney, brain, and testis (Figure 1A). Similarly, we confirmed by human multi‐tissue RT‐PCR, that *MRNIP* was expressed in several tissues with the most prominent expression in the brain, testes, and uterus (Figure 1B). *Mrnip* gene expression is present in postnatal (P) day 5 testes and onwards as shown by RT‐PCR of mouse testes at different time points (Figure 1C). The UniProt database features one mouse MRNIP protein (Q9D1F5). It is composed of 335 amino acids and has a molecular weight of 36.9 kDa. It is predicted to be an intrinsically disordered protein and based on the MobiDM database, disordered content is 50.1%,^47^ with nuclear localization, and one phosphorylation site at Ser100. No domains, repeats, motifs, or features could be predicted with confidence in mouse MRNIP protein using the Simple Modular Architecture Research Tool (SMART).^48^, ^49^

### 3.2 MRNIP is expressed in meiotic spermatocyte nuclei at the mid‐pachytene through diplotene stages

To identify the pattern and the cell type expressing MRNIP we performed double immunolabeling with MRNIP and SYCP3 antibody staining of P0, P5, P12, P15, and adult testes (Figures S1A–E″ and 1D–G‴). Staining showed that MRNIP expression was not present in testes at P0 (Figure S1A–A″), whereas in P5 and P12 testes, MRNIP‐positive staining was detected in the spermatogonia cell nucleus (Figure S1B–C″). Interestingly, at P15, when cells have started entering the pachytene stage, MRNIP expression was mainly present in the meiocyte nucleus and in occasional spermatogonia cells (Figure S1D–D″). In adult testes MRNIP staining was associated with meiocytes (Figures S1E–E″ and 1D–G‴). Next, we determined at what stage spermatocytes express MRNIP. SYCP3 is expressed on lateral axial elements in spermatocytes through most of prophase I. MRNIP and SYCP3 staining (Figure 1D–E‴) showed colocalization from pachytene stage spermatocytes (Figure 1E′–E‴, white arrows) but not with meiocytes in earlier stages (Figure 1E′–E‴, blue arrows). Next, we did immunostaining with MRNIP and Histone 1T (H1T), a meiosis‐specific histone marker of mid‐pachytene through round spermatid stages^50^ (Figure 1F–G‴). The staining showed that MRNIP expression (Figure 1G′,G‴) colocalized with H1T (Figure 1G″,G‴) from its initial expression in mid‐pachytene and diplotene spermatocytes (Figure 1F‴,G‴, white arrows). H1T continued to be expressed in round spermatids (Figure 1G″, blue arrows) whereas MRNIP expression did not (Figure 1G′–G‴, blue arrows). Taken together, our data show that MRNIP starts expression in spermatogonia cells during the first wave of spermatogenesis and after the appearance of meiocytes at P15 it becomes enriched in mid‐, late‐pachytene, and diplotene stage meiocytes.

### 3.3 MRNIP expression partially overlaps with the sex body

**FIGURE 2:**
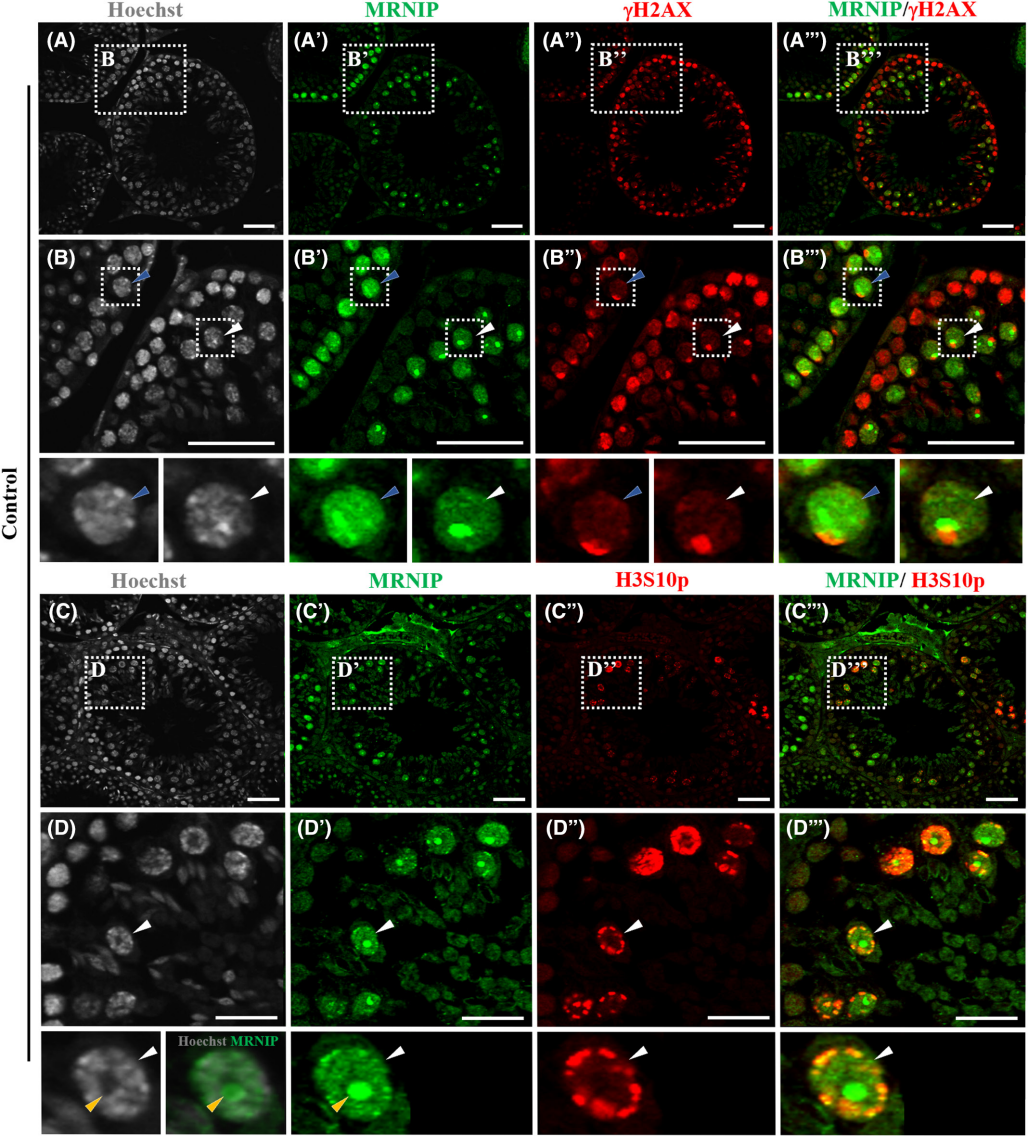
(A–B‴) MRNIP expression (green) in relation to γH2AX (red) staining in adult testes. (B–B‴) Inserts of larger magnification. (A, B) Hoechst staining depicting nuclei. (B′–B‴) MRNIP staining (green) appears as diffused and as forming droplet‐like assemblies in the mid‐pachytene stage cells and does not colocalize with γH2AX (B‴, blue arrows). Later, in diplotene stage cells MRNIP staining (green) accumulates to one droplet‐like condensate which partially overlaps with the sex body (B–B‴ and bottom inserts, white arrows). (C–D‴) Immunostaining showing condensed MRNIP (green) expression in relation to H3S10p (red) staining. (D–D‴) Inserts of larger magnification. (C, D) Hoechst staining depicting nuclei (white arrow) with the void deprived of Hoechst staining coinciding with MRNIP staining (bottom inserts, yellow arrows). (D′–D‴) MRNIP (green) droplet‐like condensate forms in diplotene stage cells depicted by H3S10p (red) antibody staining (D′–D‴ and bottom inserts, white arrows). Scale bars (A–C‴) 40 μm, (D–D‴) 20 μm.

To identify the sub‐nuclear localization of MRNIP during meiosis, we performed immunolabeling with nuclear dye Hoechst, MRNIP, and γH2AX antibodies. γH2AX is a marker of the DNA damage response at DNA double‐strand breaks in the early stages of meiosis, and later specifically labeled sex body chromatin^51^, ^52^, ^53^ (Figure 2A–B‴). The staining showed that in the mid‐pachytene stage when MRNIP expression commences it is spread through the nucleus and forms droplet‐like accumulations but does not have a distinct interaction with the sex body labeled by γH2AX at this stage (Figure 2B′–B‴, blue arrows). During late‐pachytene and diplotene, MRNIP staining condenses to one large droplet‐like assembly which partially overlaps with sex body chromatin (Figure 2B′–B‴, white arrows, overlapping can be seen in yellow B‴). To confirm that MRNIP expression associates with sex body chromatin in the diplotene stage we performed double immunolabeling with MRNIP and Phospho‐Histone H3 phosphorylation at serine 10 (H3S10p) which labels chromatin of meiotic spermatocytes in prophase I, diplotene, and metaphase where it is associated with chromosome condensation and is not detected afterward in spermatids.^54^, ^55^, ^56^ We observed that indeed, MRNIP's condensed droplet‐like appearance was present in diplotene staged cells labeled by H3S10p (Figure 2D′–D‴, white arrows). Additionally, we have noted that a void in nuclear Hoechst staining, was colocalizing with MRNIP droplet‐like condensate in the diplotene stage (Figure 2D–D′ and bottom inserts, yellow arrows). This demonstrates that initially MRNIP is spread through the nucleus in mid‐pachytene. Later droplet‐like accumulations are combining and form intense condensate which becomes partially associated with sex body chromatin in diplotene.

### 3.4 *Mrnip* knockout male mice are sterile

**FIGURE 3:**
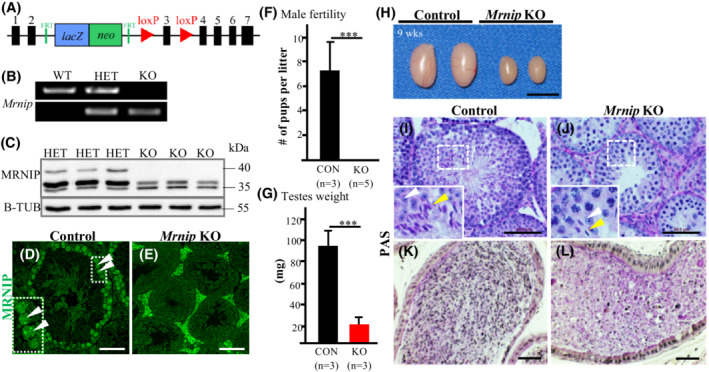
*Mrnip* knockout mice. (A) Schematic representation of the *Mrnip* ^*−/−*^ allele. Black boxes indicate coding alleles. (B) PCR genotyping of *Mrnip* wild‐type (WT), heterozygous (HET), and knockout (KO) mice. (C) Western blot with the anti‐MRNIP antibody of heterozygous (HET), and knockout (KO) mice testis lysates. Β‐TUBULIN was used as a control, three mice of each genotype were analyzed. (D, E) MRNIP antibody staining showing MRNIP‐positive cells in control (D) and no MRNIP‐positive cells in *Mrnip* ^−/−^ testes (E). (F) The average number of pups born to control mice compared to no pups in *Mrnip* ^*−/−*^ indicates that *Mrnip* ^*−/−*^ males are sterile. (G, H) Testes' weight (G) and size (H) were significantly reduced in adult *Mrnip* ^*−/−*^ mice. (I, J) PAS‐stained control (I) and *Mrnip* ^*−/−*^ (J) testis sections. (I, J inserts) White arrows point to round spermatid cells and yellow arrows point to elongating spermatids. (K, L) PAS‐stained epididymis cross‐sections of control (K) and *Mrnip* ^*−*/−^ (L). ***p < .001, Student's *t*‐test. Scale bars (D, E, I, J) 50 μm, (H) 0.5 cm, and (K, L) 100 μm. Control (CON), and knockout (KO).

To examine the function of *Mrnip* in vivo, we obtained and analyzed a knockout mouse model for *Mrnip* gene *3010026O09Rik* ^*tm1a(EUCOMM)Wtsi*^ (herein referred to as *Mrnip* ^*+/−*^ for heterozygous and *Mrnip* ^*−/−*^ for homozygous) from the Knockout Mouse Project (KOMP, Figure 3A). The *Mrnip* transgenic mice contain a *LacZ* reporter inserted between exons 2 and 3 under the control of the endogenous *Mrnip* promoter (Figure 3A). Additionally, the third exon is flanked by loxP sites (Figure 3A).

We verified the *Mrnip* gene deletion by genotyping (Figure 3B), protein production ablation by Western blot (Figure 3C), and immunohistochemistry using a MRNIP specific antibody (Figure 3D,E).

To examine the fertility of the male mice, heterozygous (HET) control and knockout *Mrnip* males were mated with wild‐type females. Control *Mrnip* ^*+/−*^ males sired 7.1 ± 2.5 (*n* = 3) pups per litter, whereas *Mrnip* ^*−/−*^ males did not sire any pups (*n* = 5, Figure 3F). The presence of vaginal plugs in females after mating with *Mrnip* ^−/−^ mice indicated that infertility was not attributed to abnormal mating behavior. The testes' weight (95.0 ± 9.7 mg vs. 24.0 ± 4.1 mg, *n* = 3, Figure 3G) and size (Figure 3H) were about four‐fold smaller in adult *Mrnip* ^−/−^ mice, whereas the body mass in controls and *Mrnip* ^*−/−*^ mice was comparable (Figure S2A). Notably, body mass, testis weight, and size were not changed in males at P15 (Figure S2A,B). Mature and motile sperm were not detected in *Mrnip* ^−/−^ mice (Movies S1 and S2). Histological analysis of PAS‐stained testes showed seminiferous tubules lacking mature spermatozoa in *Mrnip* ^−/−^ as compared to the control (Figure 3I,J). Occasionally, round and elongating spermatids were observed in *Mrnip* ^−/−^ testes (Figure 3J, insert). However, mature sperm were missing in the epididymis of *Mrnip* ^−/−^ mice as compared to control mice (Figure 3K,L, Movies S1 and S2).

### 3.5 MRNIP is a sexually dimorphic meiosis marker

Since *Mrnip* gene expression was detected in the ovaries and uterus (Figure 1A) by RT‐PCR, we tested female fertility as well. Heterozygous control and *Mrnip* ^−/−^ females were mated with wild‐type males. Our results showed that *Mrnip* ^−/−^ female fertility was normal. The control and *Mrnip* ^−/−^ females had comparable litter sizes, 7.1 ± 2.46 (*n* = 3) and 6.7 ± 2.3 (*p* = .67, *n* = 4), respectively (Figure S2C). Additionally, we performed qRT‐PCR to evaluate *Mrnip* expression in embryonic day (E) 12.5, E13.5, E16.5, newborn, and adult ovaries and testes (Figure S2D). Our analysis has demonstrated that expression was very low in embryonic testes and ovaries as compared to the expression in the adult testes, in line with the unaffected fertility rate in *Mrnip* ^−/−^ females. The immunostaining of E18.5 ovaries showed the presence of meiocytes detected by SYCP3 antibody, but no MRNIP positive cells (Figure S2E–E″, white arrows). This suggests that MRNIP is a sexually dimorphic meiosis marker specific to male meiosis.

### 3.6 Spermatocytes are lost from diplotene onwards in *Mrnip* knockout testes

**FIGURE 4:**
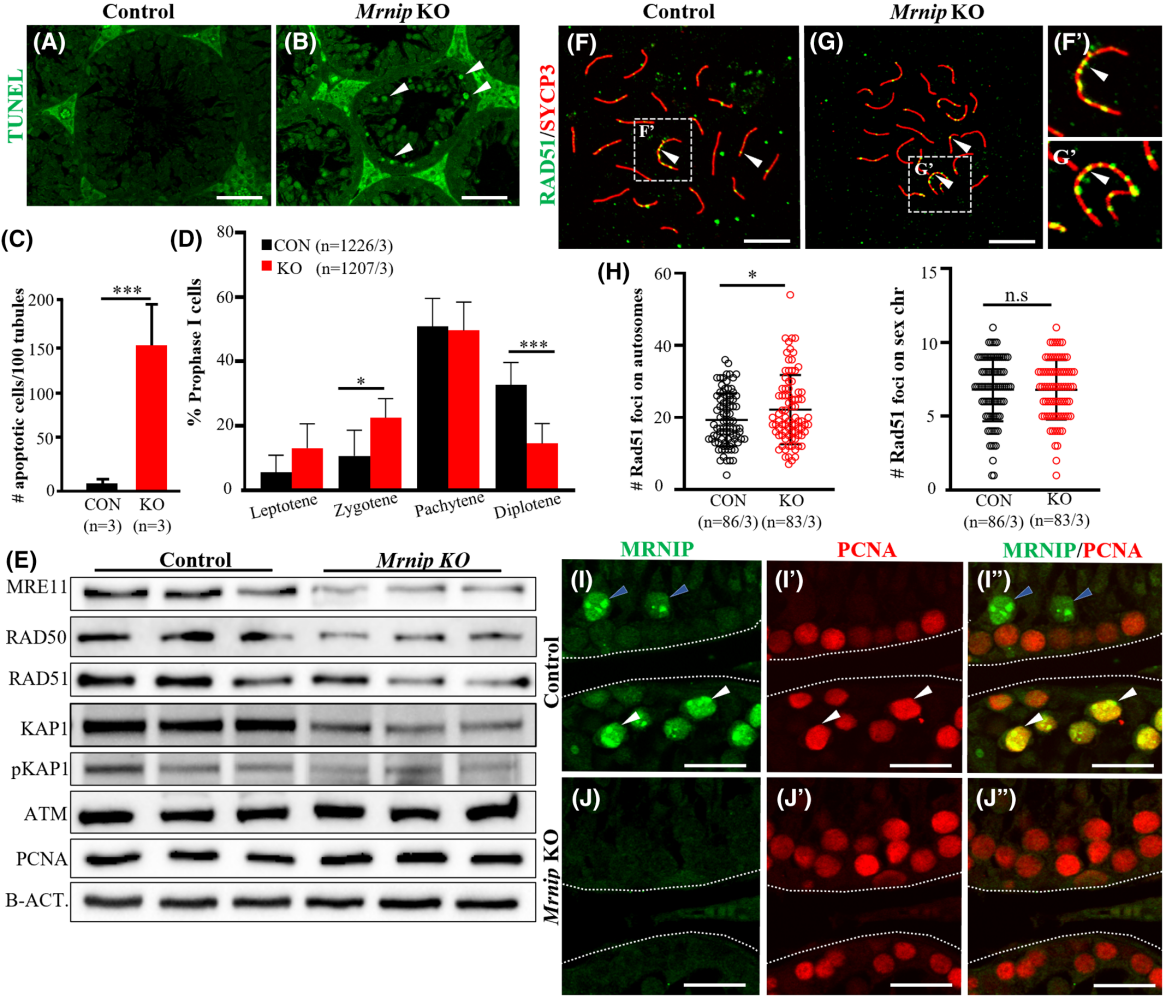
(A, B). Representative figures of TUNEL assay (green) in control (A) and *Mrnip* ^*−/−*^ testes (B, white arrows point to apoptotic cells). (C) Histogram comparing the average number of apoptotic cells per 100 seminiferous tubules from control (*n* = 3) and *Mrnip* ^*−/−*^ (*n* = 3) testes. (D) Quantification of prophase I nuclear spreads pointing to a reduced population of diplotene cells, (CON: *n* = 1226/3; KO: *n* = 1207/3). (E) Western blot analysis of multiple proteins involved in DNA DSB repair indicating reduced expression for MRE11, RAD50, RAD51, KAP1, pKAP1, and no notable difference of ATM and PCNA protein expression in control and *Mrnip* ^*−/−*^ testes lysates. B‐ACT was used as a loading control, and three mice of each genotype were analyzed. (F–G′) Nuclear spreads of control (F) and *Mrnip* ^*−/−*^ (G) chromosomes labeled with SYCP3 (red) and RAD51 antibodies (green), white arrows depicting RAD51 foci. (F′) and (G′) insert showing enlarged sex chromosomes of control (F′) and *Mrnip* ^−/−^ (G′), respectively. (H) Quantification of nuclear spreads showing a moderate significant difference in RAD51 foci counts on autosomes but not on sex chromosomes in *Mrnip* ^−/−^, (CON: *n* = 86/3; KO: *n* = 83/3). (I–J″) Testis cross‐sections stained with MRNIP (green) and PCNA (red) show colocalization of MRNIP and PCNA expression in mid‐pachytene stage spermatocytes (I–I″, white arrows) but not in later stages (I, I″, blue arrows). (J–J″) Micrographs depict undisturbed PCNA expression in *Mrnip* ^*−/−*^ testes. Statistical analysis: *p < .05, ***p < .001, Student's *t*‐test, ±SD. Scale bars (A, B) 20 μm, (F, G) 10 μm, (I–J″) 20 μm. Control (CON) and knockout (KO).

As *Mrnip* ^*−/−*^ testes had reduced seminiferous tubule size due to missing cell populations in the later stages of spermatogenesis, we performed a TUNEL assay to identify the apoptosis rate in control and *Mrnip* ^*−/−*^ testes. Control seminiferous tubules had occasional TUNEL positive cells whereas *Mrnip* ^*−/−*^ showed a high frequency of apoptotic cells (Figure 4A,B) in immunostaining and was confirmed by quantification (Figure 4C). To define at what time and which cells were lost in knockout testes, we quantified prophase I cells in nuclear spreads. This indicated that there was a trend of higher counts of leptotene and zygotene stage cells in *Mrnip* ^*−/−*^, whereas pachytene stage cell counts were comparable in control and *Mrnip* ^*−/−*^. However, diplotene stage spermatocyte numbers were critically reduced in *Mrnip* ^*−/−*^ testes (Figure 4D).

### 3.7 DNA DSB repair protein expression: MRE11, RAD50, RAD51, KAP1, and pKAP1, were downregulated, but not ATM or PCNA in *Mrnip* ^*−/−*^ testes

In previous studies, MRNIP was shown to be involved in DSB repair.^33^, ^34^ Given this, we analyzed the expression levels of DSB repair proteins, MRE11, RAD50, and Rec A homolog RAD51. The Western blot analysis demonstrated that the expression of all three of these DSB repair proteins was diminished in the *Mrnip* ^−/−^ whole testes lysates as compared to heterozygous controls (Figure 4E). The decreased expression levels of DSB repair proteins raised the question if protein localization and DSB repair were altered in *Mrnip* ^−/−^ mice. In the case of MRE11 expression, localization was comparable in *Mrnip* ^−/−^ to control specimens in testis cross‐sections (Figure S3A–B‴). The quantification of early and mid‐pachytene stage RAD51 positive foci in autosomes showed a moderate, however, a significant increase of RAD51 foci in *Mrnip* ^−/−^ (mean foci 22.19 ± 9.6, *n* = 83) nuclear spreads in comparison to control (mean foci 19.35 ± 7.34, *n* = 86, *p* = .032, Figure 4F–H) nuclear spreads. The RAD51 foci counts were similar in sex chromosomes in control (mean foci 6.81 ± 2.15, *n* = 86) and *Mrnip* ^−/−^ (mean foci 6.80 ± 2.02, *n* = 83, *p* = .95, Figure 4F′–H). This suggests a tendency for unrepaired DSBs in autosomes but not in sex chromosomes during meiosis in *Mrnip* ^−/−^ testes.

Next, we analyzed the KRAB‐associated protein 1 (KAP1), which controls DNA repair in heterochromatin,^57^ and which was shown to be affected by the lack of MRNIP in earlier in vitro studies with human cells treated with ionizing radiation.^33^ Our Western blot analysis has shown that the expression of both KAP1 and phosphorylated KAP1 (pKAP1) were decreased in *Mrnip* ^−/−^ (Figure 4E). KAP1 also serves as a substrate of another major protein involved in the DSB repair pathway, the ATM protein.^9^, ^57^ Since *Mrnip* deletion has affected ATM substrates' expression (KAP1 and pKAP1), we have analyzed if ATM expression was influenced. The analysis by Western blot showed no clear changes in ATM expression in response to *Mrnip* deletion in testis lysate (Figure 4E).

We further investigated potentially abnormal synapsis in pachytene spermatocytes by co‐immunostaining with SYCP1 and SYCP3 antibodies. Analysis and quantification showed that most of the control (95.3% ± 4.63, *n* = 181) and *Mrnip* ^*−/−*^ (95.6% ±3.96, *n* = 195, *p* = .75) autosomes were aligned, whereas sex chromosomes were still unsynapsed as expected both in control and *Mrnip* ^−/−^ (Figure S3C–E). The data suggest that synapsing is likely not affected in *Mrnip* ^−/−^ meiocytes.

In an earlier study, it was shown that MRNIP plays a role as a replication fork protection factor during DNA DSB in irradiated somatic cells.^34^ Based on this, we raised the question if MRNIP plays a similar role during male meiosis and analyzed fork‐associated proliferating cell nuclear antigen (PCNA) expression. PCNA plays an essential role in DNA replication, synthesis, repair, and cell cycle control, and it has been used as an indicator of cells undergoing DNA synthesis and repair. PCNA reflects a function of DNA excision repair in meiosis.^58^, ^59^ Our Western blot analysis did not show notable changes in the expression levels of PCNA in control and *Mrnip* ^−/−^ whole testes lysates (Figure 4E). PCNA immunostaining is generally observed in mitotically proliferating spermatogonia and spermatogenic cells in meiotic prophase, specifically in zygotene and pachytene stage spermatocytes undergoing meiotic recombination.^60^ Our immunostaining demonstrated that PCNA was co‐expressed with MRNIP only in mid‐pachytene cells, but not before or after (Figure 4I–I″). The immunostaining showed that PCNA expression was comparable in control and *Mrnip* ^−/−^ testes (Figure 4I–J″). To rule out the possibility that PCNA was staining only earlier stage meiocytes which would be MRNIP negative, we performed additional double labeling with H1T, labels mid‐pachytene to round spermatid stage cells, and PCNA antibodies (Figure S3F–G″). The immunostaining showed that PCNA was expressed not only in early but also in mid‐ to late‐pachytene stage meiocytes co‐labeled with H1T and PCNA antibodies in control and *Mrnip* ^−/−^ (Figure S3F–G″, white arrows).

### 3.8 Meiotic sex chromosome inactivation is impaired in *Mrnip* ^−/−^ spermatocytes

**FIGURE 5:**
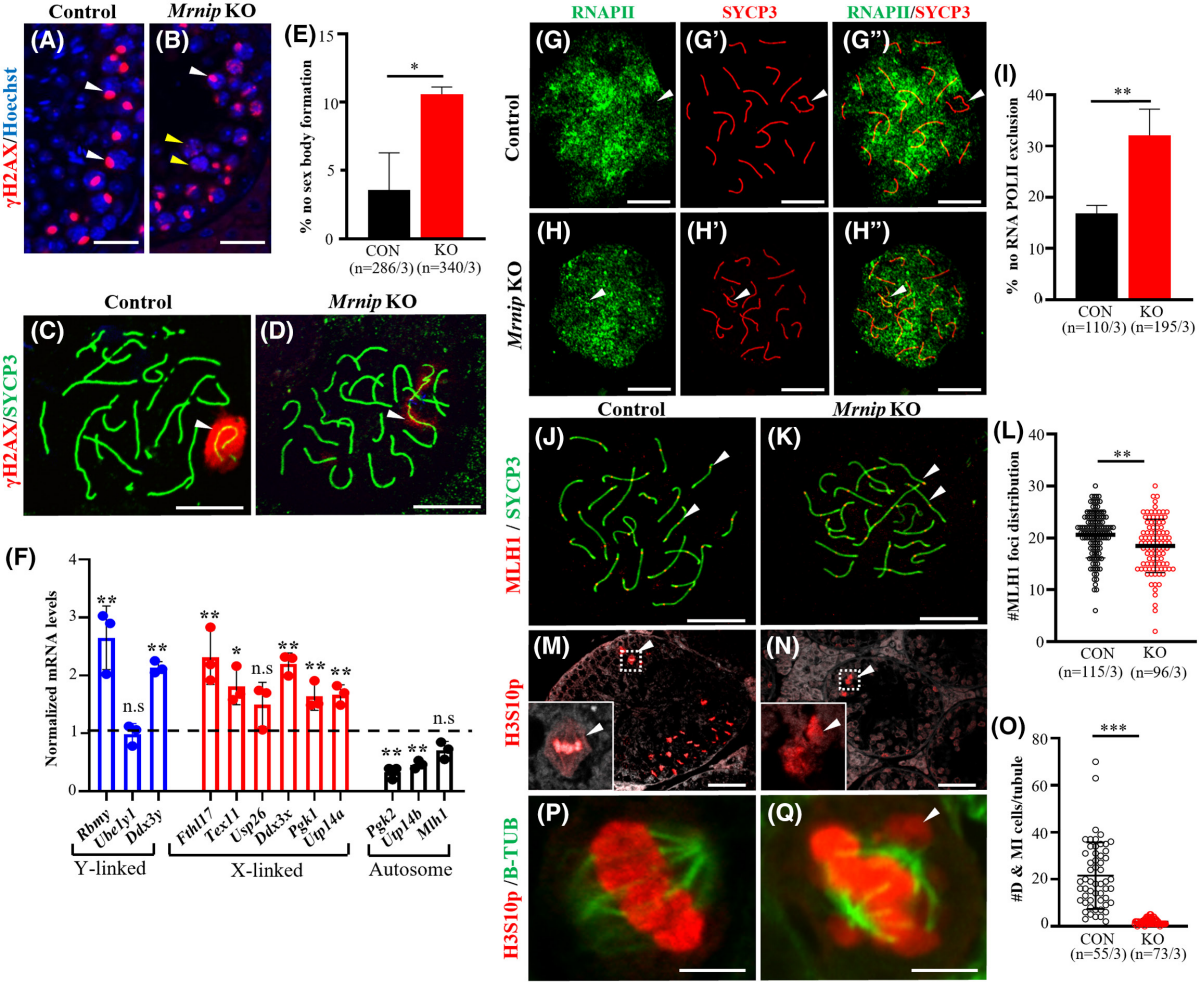
(A, B) Immunostaining showing γH2AX expression in sex bodies (A, B white arrows) in *Mrnip* ^+*/−*^ and defects in sex body formation in *Mrnip* ^*−/−*^ (B, yellow arrows) testis cross‐sections. Nuclear spreads of control (C) and *Mrnip* ^*−/−*^ (D) spermatocytes. Chromosomes are labeled by SYCP3 (green) and sex bodies encapsulating sex chromosomes (white arrows) labeled by γH2AX. (E) Quantification of nuclear spreads with and without sex bodies in control (*n* = 286/3) and *Mrnip* ^*−/−*^ (*n* = 340/3). (F) QRT‐PCR analysis of genes known to be inactivated by MSCI, expressed on the X and Y chromosomes and genes expressed on autosomes in *Mrnip* ^*−/−*^ normalized with *Gapdh* and compared to control (dashed line). (G–H″) Meiotic spreads of control (G–G″) and *Mrnip* ^*−/−*^ (H–H″) spermatocytes labeled with SYCP3 (G′, H′, red) and RNA Pol II (G, H, green) showing RNA Pol II exclusion from sex chromosome in control (G–G″, white arrows) but not in *Mrnip* ^*−/−*^ spermatocytes (H–H″, white arrows). (I) Quantification of RNA Pol II exclusion show that 32.1% of pachytene spermatocytes retain staining around XY chromosomes in *Mrnip* ^*−/−*^ mice (CON: *n* = 110/3; KO: *n* = 195/3). (J, K) Nuclear spreads labeled with SYCP3 (green) and MLH1 foci (red, white arrows) in control (J) and *Mrnip* ^*−/−*^ (K). (L) Quantification shows a decrease of MLH1 foci count in *Mrnip* ^*−/−*^ (CON: *n* = 115/3; KO: *n* = 96/3). (M, N) H3S10p immunolabeling in control (M) and *Mrnip* ^*−/−*^ (N) testes, white arrows point to meiotic spermatocytes in prophase I diakinesis and metaphase. (O) Quantification of H3S10p‐positive cells in diakinesis (D) and metaphase I (MI) per seminiferous tubule cross‐section in control and *Mrnip* ^*−/−*^ showing a decreased number of H3S10p‐positive cells in *Mrnip* ^*−/−*^ (CON: *n* = 55/3; KO: *n* = 73/3). (P, Q) H3S10p and B‐TUBULIN staining in control (P) and *Mrnip* ^*−/−*^ (Q, arrows point to unaligned chromosomes). Statistical significance: n.s not significant, *p < .05, **p < .01, ***p < .0001, Student's *t*‐test, ±SD. Scale bars (A, B) 20 μm, (C, D, G–H″ J, K, P, Q) 10 μm, (M, N) 50 μm. Control (CON) and knockout (KO).

Immunostaining of testis cross‐sections stained with γH2AX, a marker of the DNA damage response at DNA double‐strand breaks in early stages of meiosis, and later specific to sex bodies^51^, ^52^, ^53^ has indicated that many *Mrnip* ^*−/−*^ meiocytes lacked sex bodies (Figure 5A,B). Aberrant γH2AX staining in sex bodies was shown to be associated with MSCI defects; given this, we explored if *Mrnip* could be involved in MSCI. We analyzed sex body formation and sex chromosome pairing in control and *Mrnip* ^*−/*−^ nuclear spreads immunostained with SYCP3 and γH2AX antibodies (Figure 5C,D). Quantification of nuclear spreads in the pachytene stage showed that sex bodies were not formed in 10.60% ±0.52 (*n* = 340) of *Mrnip* ^*−/−*^ as compared to 3.58% ±2.71 of control (*n* = 286) spermatocytes (*p* = .02, Figure 5E). We analyzed X and Y chromosome pairing in the meiocytes that formed the sex body. The quantification showed that the X and Y chromosome pairing was normal, and the counts of paired X and Y chromosomes were comparable between control 91.3% ±5.27 (*n* = 287) and *Mrnip* ^*−/−*^ 90.15% ±4.93 (*n* = 329) when the sex body was formed.

To further explore *Mrnip*'s role in MSCI, we performed qRT‐PCR of genes located specifically on X and Y chromosomes using adult testis cDNA. We compared the expression of Y‐linked (*Rbmy*, *Ube1y1, Ddx3y*) and X‐linked (*Fthl17*, *Tex11, Usp26, Ddx3x, Pgk1*, and *Utp14a*) genes that were shown to be associated with MSCI, and autosomal (*Pgk2, Utp14b*, and *Mlh1*) genes as controls.^20^, ^31^, ^61^, ^62^, ^63^ *Mlh1* gene (Chr 9) expressed in germ cells served as an indicator that cells had an overall non‐reduced transcription. Our data showed that most of the analyzed X‐ and Y‐linked genes expression remained active in *Mrnip* ^−/−^ mice (except for *Usp26* and *Ube1y1*, Figure 5F), but were inactivated in control, suggesting a role of *Mrnip* in MSCI.

MSCI in male germ cells is partially possible due to the retroposition of X‐linked genes to autosomes. During this process, genes become duplicated, and genes expressed on the male X chromosome become silenced and their counterpart on autosomes start to be expressed.^20^, ^21^, ^27^, ^62^, ^64^ Loss of function of retrogenes such as *Pgk1/Pgk2* and *Utp14a*/*Utp14b* has been shown to cause spermatogenic arrest.^62^, ^64^

We have further analyzed if insufficient silencing of X‐linked genes affected autosomal gene de‐repression. Inactivation of genes expressed on sex chromosomes has been shown to trigger a compensatory de‐repression of related retrogenes on autosomal chromosomes during MSCI.^20^, ^62^, ^64^ Such examples include housekeeping genes *Pgk1* and *Utp14a*, when they are silenced, the autosomal genes *Pgk2* and *Utp14b* accordingly, become de‐repressed and serve as functional backups for their inactivated counterparts located on the X‐chromosome.^20^, ^62^ Our data showed that *Pgk1* and *Utp14a* gene silencing was not taking place in *Mrnip* ^*−/−*^ testes and consequently, *Pgk2* and *Utp14b* remained inactive (Figure 5F) suggesting additional defects associated with inadequate MSCI.

RNA Polymerase II (RNA Pol II) exclusion is observed in non‐transcribing sex bodies and inappropriate exclusion is often associated with defective MSCI.^20^, ^31^, ^65^, ^66^ To further investigate the role of *Mrnip* in MSCI we performed immunostaining of meiotic spreads using antibodies against SYCP3 and RNA Pol II (Figure 5G–H″). Quantification showed that RNA Pol II was not excluded in 32.1% ±5.08 of *Mrnip* ^*−/−*^ pachytene spermatocytes (*n* = 195) as compared to the control (*n* = 110, *p* = .0076, Figure 5I, white arrows). Altogether our results showed that more *Mrnip* ^*−/−*^ meiocytes had ablated sex body formation, insufficient inactivation of multiple sex chromosome‐specific genes, along with inadequate de‐repression of retrogenes on autosomes and reduced exclusion of RNA Pol II from sex body indicating defective MSCI in *Mrnip* ^*−/−*^.

### 3.9 Cross‐over formation is altered whereas the cells that survive through earlier meiotic checkpoints form bivalents

We showed that MRNIP and H1T expression coincide during the mid‐pachytene to diplotene stage in spermatocytes (Figure 1G–G‴). We also observed that some diplotene and later stage cells were detected in *Mrnip* ^−/−^ testes, however very few, as evidenced by H1T antibody staining (Figure S4A,B). This encouraged us to study surviving cells to identify damages occurring due to *Mrnip* deletion. We questioned if recombination was affected in these cells. MutL homolog 1 (MLH1) localizes to the cross‐over sites in meiotic chromosomes.^19^, ^67^ It is presumed that meiotic cells gain competence to enter metaphase after crossing‐over. MLH1 foci are first assembled onto chromosomes during pachytene^19^, ^67^ which coincides with MRNIP expression in meiotic spermatocytes. Given this, we analyzed the number of MLH1 foci per nuclei in nuclear spreads of control and *Mrnip* ^*−/*−^ cells (Figure 5J,K). The quantification revealed a significant decrease of MLH1 foci in *Mrnip* ^*−/−*^ (mean foci 18.46 ± 5.13, *n* = 96) in comparison to control (mean foci 20.63 ± 4.47, *n* = 115, *p* = .0012, Figure 5L).

Phospho‐Histone H3 phosphorylation at serine 10 (H3S10p) labels chromatin of meiotic spermatocytes in prophase I diplotene and metaphase where it is associated with chromosome condensation and is not detected afterward in spermatids.^54^, ^55^, ^56^ Immunolabeling showed that *Mrnip* ^*−/*−^ testes displayed fewer cells bearing the H3S10p marker (Figure 5M,N). Further quantification of H3S10p staining of pro‐metaphase (diakinesis) and metaphase cells has confirmed a significant reduction of H3S10p‐positive spermatocytes in *Mrnip* ^*−/*−^ testes (1.95 ± 1.2, *n* = 73) as compared to the control (21.55 ± 14.17, *n* = 55, *p* = .0001) (Figure 5O) as expected. The presence of typical metaphase events such as the phosphorylation of Histone H3 at serine 10 (H3S10p) in *Mrnip* ^−/−^ spermatocytes suggested that there is no inhibition in the onset of meiotic metaphase for the cells that have made it through the meiotic silencing of sex chromosomes checkpoint. However, the condensed chromosomes often failed to align correctly onto the spindle apparatus in *Mrnip* ^−/−^ spermatocytes as indicated by H3S10p and B‐TUBULIN double‐immunostaining (Figure 5P,Q, arrows point to unaligned chromosomes).

Univalent chromosomes result in the activation of checkpoints leading to spermatocyte apoptosis. To see if *Mrnip* ^*−/−*^ chromosomes form bivalents, we quantified the presence of univalent and bivalent chromosomes in metaphase I spreads in control and *Mrnip* ^*−/−*^ testes (Figure S4C–F). The analysis did not show significant differences in bivalent versus univalent counts per cell between the controls and knockouts (Figure S4G), indicating that the cells that survive through earlier meiotic checkpoints do form bivalents in *Mrnip* ^*−/−*^ testes.

### 3.10 DNA DSBs and synaptonemal complex transverse elements precede MRNIP expression

**FIGURE 6:**
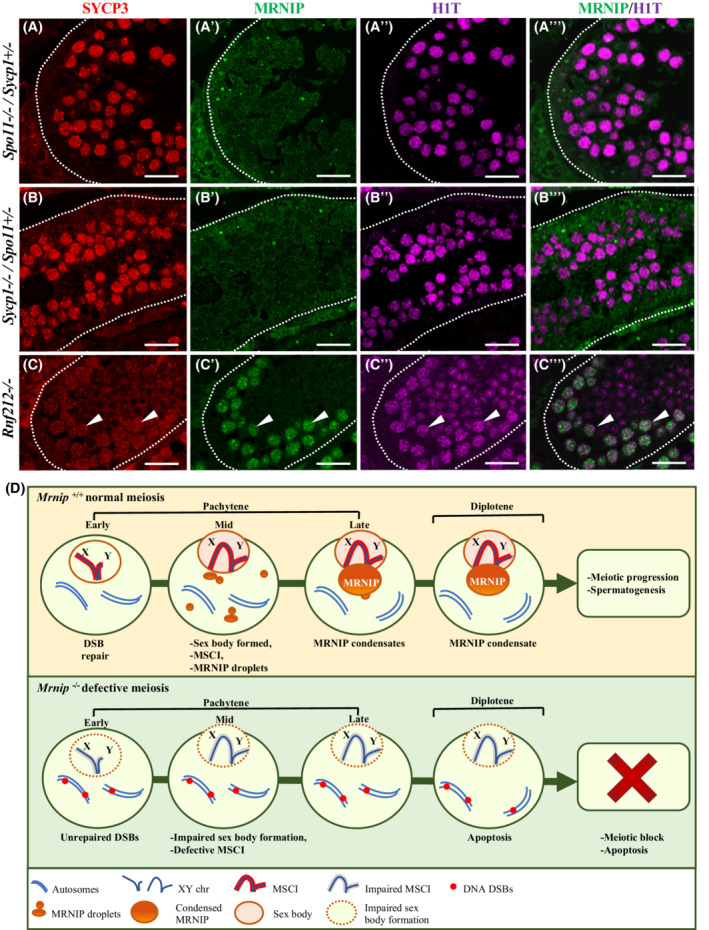
(A–A‴) MRNIP was not expressed in *Spo11* ^*−/−*^ mouse testes, compare SYCP3 (A, red), MRNIP (A′, green), H1T (A″, magenta), and merged (A‴) micrographs. (B–B‴) MRNIP was not expressed in *Sycp1* ^*−/−*^ mouse testes, compare SCYP3 (B, red), MRNIP (B′, green), H1T (B″, magenta), and merged (B‴) micrographs. H1T expression in *Spo11* ^*−/−*^/ Scyp1^−/−^ indicates the presence of mid‐pachytene spermatocytes. (C–C‴) MRNIP expression was not affected in *Rnf212* ^−/−^ mouse testes. Immunostaining shows MRNIP colocalizes with H1T in spermatocytes at the mid‐pachytene to diplotene stage. Compare SYCP3 (C, red, white arrows), MRNIP (C′, green, white arrows), H1T (C″, magenta, white arrows), and merged (C‴, white arrows) micrographs. Scale bar (A–C‴) 20 μm. (D) Graphical representation of MRNIP expression's subnuclear localization and defects noted in the case of *Mrnip* deletion. MRNIP expression starts in mid‐pachytene as diffused multiple droplet‐like condensates. By diplotene, they accumulate to one large assembly that partially overlaps with the sex body in wild‐type meiocytes. In the case of *Mrnip* deletion, DNA DSB repair efficacy is impaired, fewer cells form sex bodies, and multiple genes associated with the MSCI remain active. This eventually leads to germ cell apoptosis, peaking in diplotene, and causing infertility in male mice.

We decided to identify what major meiotic events are required to precede MRNIP expression. *Spo11* is one of the essential DNA cleavage mediators that forms DSBs that initiate meiotic recombination.^5^ We used *Spo11* ^*−/−*^ */Sycp1* ^*+/−*^ mouse testes to assess the importance of DNA double‐strand break formation during the onset of meiosis for MRNIP expression (Figure 6A–A‴). Triple labeling with MRNIP, H1T and SYCP3 antibodies showed that even though mid‐pachytene cells are present in *Spo11* ^*−/−*^ mice, MRNIP is not expressed in the absence of *Spo11* (Figure 6A–A‴).

We next analyzed the effect of synapsis on MRNIP expression by examining MRNIP in the absence of Synaptonemal complex protein 1 (SYCP1), a transverse axial element protein of the synaptonemal complex,^68^ in *Sycp1* ^*−/−*^/*Spo11* ^*+/−*^ mouse testes. The immunostainings with MRNIP, H1T, and SYCP3 antibodies showed that mid‐pachytene cells were present in *Sycp1* mutant mice, whereas MRNIP was not expressed in *Sycp1* ^*−/−*^ testes (Figure 6B–B‴).

We also examined whether the defective cross‐overs affect MRNIP expression by using mice carrying a deletion of Ring finger protein 212 (*Rnf212*), a SUMO ligase required for crossing‐over formation.^39^, ^69^ Immunostainings with MRNIP, H1T, and SYCP3 antibodies determined that the MRNIP expression was not altered in *Rnf212* ^*−/−*^ testes and that MRNIP colocalizes with H1T in mid‐pachytene to diplotene stage spermatocytes (Figure 6C–C‴).

This data suggest that DNA DSBs and the presence of synaptonemal complex transverse elements precede MRNIP expression, further suggesting the role of MRNIP in the later stages of meiotic prophase I.

## 4 DISCUSSION

Herein, we show that *Mrnip* mRNA is expressed in multiple tissues and enriched in the testes. MRNIP expression is initiated at P5 in spermatogonia, it is not detected in leptotene and zygotene stages, and becomes pronounced in spermatocyte nuclei during mid‐pachytene and continues through the diplotene stage of prophase I in mice. We showed that, in juvenile testes, prior to meiosis initiation, MRNIP expression was associated with spermatogonia. This goes in line with the single‐cell RNA sequencing data of juvenile mouse testes at P1.5, P3.5, and P5.5 depicting *Mrnip* expression in germ cells but not in somatic cell populations.^70^ We demonstrated that in adult testes, MRNIP expression dominates in the mid‐pachytene to diplotene meiocytes. However, it becomes difficult to distinguish if spermatogonia in adult testes continue expressing MRNIP solely from immunostainings. The single‐cell transcriptome analysis by Hermann and colleagues identified *Mrnip* mRNA transcripts in spermatogonia, but not in leptotene/zygotene stage spermatocytes, and the peak expression in pachytene/diplotene cell populations is supported by our findings.^71^ This data suggest that MRNIP likely continues to be expressed in adult spermatogonia as well, but very weakly, as shown by immunostainings.

Our study of the sub‐nuclear MRNIP expression shows that first, as MRNIP expression starts in mid‐pachytene meiocytes it is spread throughout the nucleus as multiple droplet‐like condensates. Later, the droplet‐like condensate counts become reduced, and their size increases. Eventually, in diplotene, MRNIP staining becomes one large droplet‐like condensate partially associated with sex body heterochromatin. Our studies show that MRNIP expression initiation and sex body formation take place in the mid‐pachytene nucleus. Whereas the MRNIP association with the sex body occurs only later in late‐pachytene and diplotene when MRNIP forms droplet‐like condensate, which partially overlaps with classical, γH2AX delineated, sex body borders. Based on this interaction timing difference, it seems MRNIP is not involved in sex body formation, but existing physical interaction could influence the MSCI efficacy.

Recent studies showed that MRNIP forms liquid‐like condensates to promote homologous recombination‐mediated DSB repair in somatic cells.^35^ Concurrently it is hypothesized that liquid–liquid phase separation, could be the mechanism behind the nucleolus, sex body, and other membrane‐less organelle formation, and MSCI during male meiosis.^72^, ^73^ This suggests that the droplet‐like MRNIP condensation observed in diplotene could form a membrane‐less nucleoli‐like organelle based on the liquid–liquid phase separation properties which would interact with the sex body heterochromatin. Interaction between nucleoli and the inactive X chromosome (Xi) was reported to occur in female cells.^74^, ^75^ Scientists showed that the Xi must continuously attend the perinucleolar compartment to maintain its inactivation.^75^ Our study suggests that such interaction could exist during male meiosis as well. Whereas further research will be needed to establish the nature and mechanism of MRNIP interaction with the sex body.

Additionally, we have shown that MRNIP expression was not detected in female meiocytes. Overall, this marks MRNIP as a specific, sexually dimorphic biomarker, for male spermatogonia and meiotic prophase I mid‐pachytene to diplotene stage spermatocytes (see a schematic timeline of MRNIP expression during meiosis in relation to other meiosis markers discussed in this study, Figure S4H).

Herein, we demonstrated that the deletion of the *Mrnip* gene causes infertility in male mice correlated with multiple abnormalities through spermatogenesis and critical loss of prophase I diplotene stage spermatocytes. Staples and colleagues have demonstrated an association between MRNIP and MRN complex proteins (MRE11, RAD50, and NBS1) during DNA DSB repair after gamma radiation in human cell lines and named the protein accordingly.^33^, ^34^ DNA DSB repair plays an essential role during meiosis and must be completed after the prophase I pachytene stage to permit the first meiotic cell division.^17^ After DNA DSB induction by SPO11, the DNA DSB repair machinery becomes activated. MRE11 and RAD50 start to be expressed during zygotene along with other DNA DSB repair proteins (ATM, RAD51, KAP1, etc.). We showed that MRNIP expression in adult testes starts in the mid‐pachytene meiocytes. Even though *Mrnip* expression starts later during meiosis, the expression levels of multiple DNA DSB repair proteins (MRE11, RAD50, RAD51, KAP1, and pKAP1) were decreased in *Mrnip* ^*−/−*^ testes lysates. Along with this, RAD51 foci counts were moderately increased in pachytene spermatocyte autosomes indicating the presence of unrepaired DNA DSBs. Given that the major DNA DSB repair events are taking place prior to MRNIP expression in spermatocytes, it is likely that during meiosis MRNIP exerts its effect on DNA DSB repair mostly through an indirect, yet uncharacterized feedback mechanism.

MLH1 protein localizes to meiotic cross‐over sites and marks the chiasmata positions.^19^, ^76^ The MLH1 foci counts were slightly decreased in *Mrnip* ^*−/−*^ *mice*. This could suggest reduced formation of chiasmata. If this were the case, then chromosome bivalent formation at meiotic metaphase would likely be disturbed, which was not the case in remaining *Mrnip* ^−/−^ meiocytes that have reached metaphase. However, even though the cells bypassed earlier meiotic checkpoints and initiated metaphase, they often fail to align on the spindle and very few cells continued differentiation in *Mrnip* ^−/−^.

We showed that PCNA and MRNIP expression during meiosis, overlapped only in mid‐pachytene stage spermatocytes, when MRNIP expression begins and PCNA expression decreases. The PCNA expression levels and patterns were not changed in *Mrnip* ^*−/−*^ testes. This implies MRNIP is not necessarily playing a crucial role as a replication fork protection factor during meiosis as it does in the case of gamma radiation induced DNA DSB repair in somatic cells.^34^

Failure in sex body formation and RNA Pol II exclusion from sex body are morphological features often associated with defects in X and Y chromatin silencing.^20^, ^31^, ^65^, ^66^ Herein, we showed that nearly every ninth spermatocyte did not form a sex body and every third failed to exclude RNA Pol II in *Mrnip* ^*−/−*^. This alone could hardly account for such drastic spermatocyte loss, whereas an association with the lethal effects of non‐repressed genes from X and Y chromosomes could explain the massive loss of post‐pachytene spermatocytes in *Mrnip* ^*−/−*^ testes. Indeed, our study revealed that many genes on X and Y chromosomes were still transcriptionally active in *Mrnip* ^*−/−*^ instead of being silenced as in controls. Additionally, we showed that retrogene repression and de‐repression were altered in *Mrnip* ^*−/−*^ testes. *Pgk1* and *Utp14a* expressed on the X chromosome remained active in *Mrnip* ^*−/−*^ testes, whereas they were already inactivated in the control. Consequently, retrogenes *Pgk2* and *Utp14b* expressed on autosomes remained silenced in *Mrnip* ^*−/−*^ testes, instead of becoming de‐repressed as in the control to balance the missing function of the repressed gene. This repression and de‐repression sequence is altered in *Mrnip* ^*−/−*^ meiocytes. The loss of function of at least one such retrogenes has been shown to lead to spermatogenic arrest.^17^, ^20^, ^21^, ^27^, ^62^, ^64^, ^77^

Accumulating studies propose that meiotic silencing involves many genes associated with the mitotic DNA damage response such as *Brca1*, *H2ax*, *Atr*, *Parp2*, *Mdc1*, *Hormad1*, *Hormad2*, and *Sycp3*.^20^, ^21^, ^28^, ^29^, ^30^, ^31^, ^32^, ^78^, ^79^

Earlier studies showed that *Mrnip* is associated with DNA DSB repair in somatic cells^33^, ^34^ and our work demonstrates *Mrnip*'s association with a role in MSCI. However, *Mrnip* does not follow the classical pattern seen in other genes associated with MSCI, where defective synapsis and stage IV pachytene meiocyte loss is seen.^17^ In *Mrnip* ^*−/−*^ synapsis was normal and this, likely, allowed meiocytes to survive through stage IV, mid‐ and late‐pachytene. This seemingly normal synapsis could be explained by the timing of MRNIP expression in meiocytes, starting at mid‐pachytene when the synapsing process and SYCP1 expression are coming to an end. The major loss of meiocytes in *Mrnip* ^*−/−*^ took place during the diplotene stage, likely due to insufficient MSCI.

There is an ongoing discussion whether the synapsis checkpoint and the MSCI checkpoint are two separate checkpoints or if MSCI is a secondary event followed by failure in synapsis.^52^, ^80^ Based on our study, where *Mrnip* knockout chromosomes synapsed normally but MSCI was faulty, we are tempted to hypothesize that they could be two separate checkpoints; however, this requires further verification. It is important to note that a recent study by Lin et al. showed a reduced count of pachytene meiocytes and unsynapsed chromosomes in *Mrnip* ^*−/−*^. However, it was difficult to judge the severity of the defect in the presented mouse model since quantification of synapsed versus non‐synapsed chromosomes was not included.^36^

Furthermore, our study identified two critical upstream genes preceding *Mrnip* expression during male meiosis. First, *Spo11*, of which expression is essential for double‐strand break formation and subsequent DNA recombination and homologous pairing during meiosis,^4^, ^5^ is required for *Mrnip* expression initiation in mouse testes. This suggests that DNA DSBs are one of the triggers necessary for *Mrnip* expression. Another critical event during meiosis is synaptonemal complex formation; specifically, *Sycp1* expression which promotes homologous recombination during meiosis.^15^, ^16^ Our data demonstrate that *Sycp1* expression is vital for the initiation of *Mrnip* expression.

In conclusion, our study shows that MRNIP is a specific spermatocyte biomarker of mid‐pachytene through diplotene stages. We demonstrate that MRNIP staining partially overlaps with the sex body in diplotene. Furthermore, we showed that MRNIP is required for proper DNA DSB repair protein function, efficient meiotic sex chromosome inactivation, and full‐fledged spermatogenesis (schematic representation of key features of *Mrnip* function during meiosis, Figure 6D). The ablation of *Mrnip* function causes infertility in male mice. Finally, the presented data raise the possibility that impaired function of *MRNIP* could be a cause of infertility in humans and other species.

## AUTHOR CONTRIBUTIONS

Martin M. Matzuk and Ramiro Ramirez‐Solis initiated the study; Renata Prunskaite‐Hyyryläinen and Julio M. Castañeda designed research; Samina Kazi, Renata Prunskaite‐Hyyryläinen, Julio M. Castañeda, Audrey Savolainen, Yiding Xu, Ning Liu, Huanyu Qiao, Kaori Nozawa, and Zhifeng Yu performed research; Samina Kazi, and Renata Prunskaite‐Hyyryläinen analyzed data; Renata Prunskaite‐Hyyryläinen and Samina Kazi, wrote the article; Renata Prunskaite‐Hyyryläinen and Martin M. Matzuk acquired grants to support the study.

## DISCLOSURES

The authors have no conflicts of interest to declare.

## Supporting information


Figure S1

Figure S2

Figure S3

Figure S4

Table S1

Table S2

Video S1

Video S2

Text S1

## Data Availability

The data that support the findings of this study are available in the methods and/or supplementary material of this article.
