# Supplementary figures and images for: MRNIP interacts with sex body chromatin to support meiotic progression, spermatogenesis, and male fertility in mice
Source: FASEB J. 2022 Aug 3;36(9):e22479. doi: 10.1096/fj.202101168RR (PMC9544956; doi:10.1096/fj.202101168RR)

Fig. S1

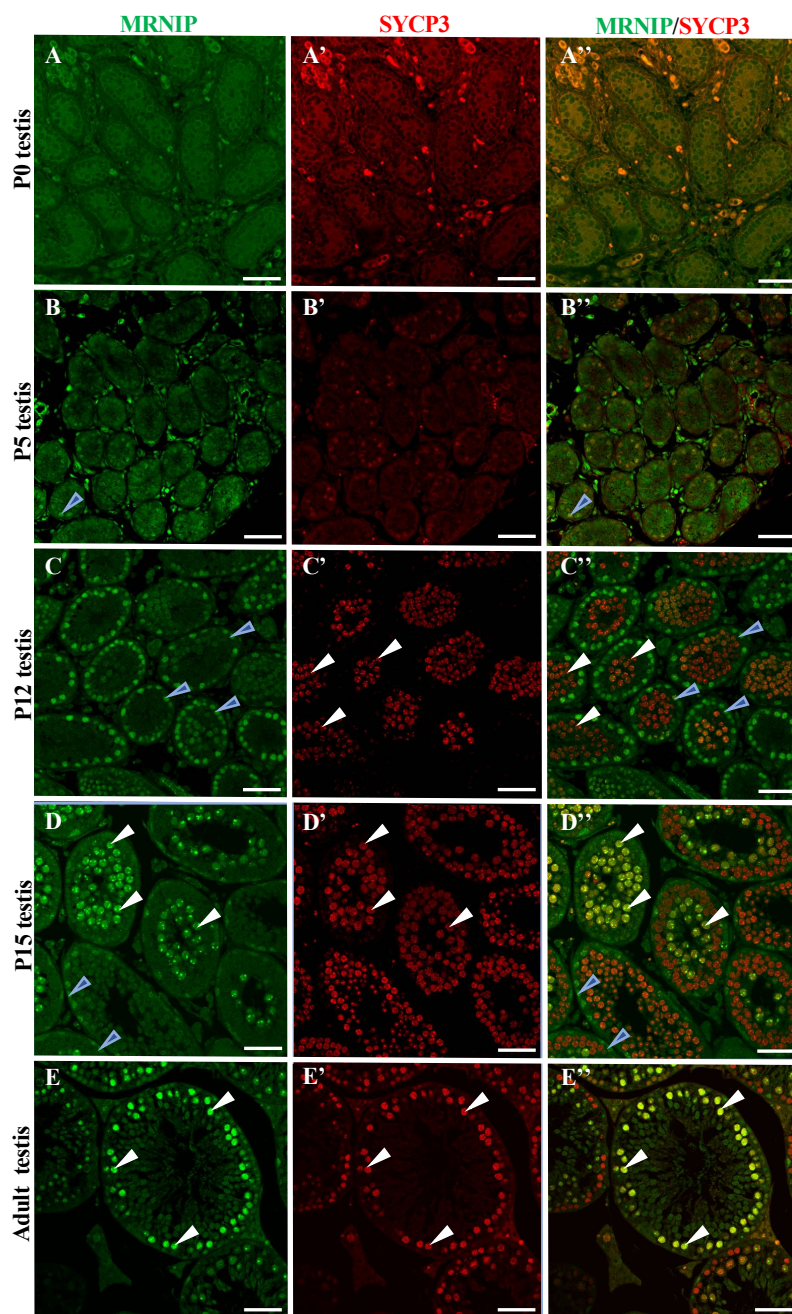

Supplement: Supplementary file 1 — Figure S1 [file FSB2-36-0-s006.pdf]

**Fig. S2**

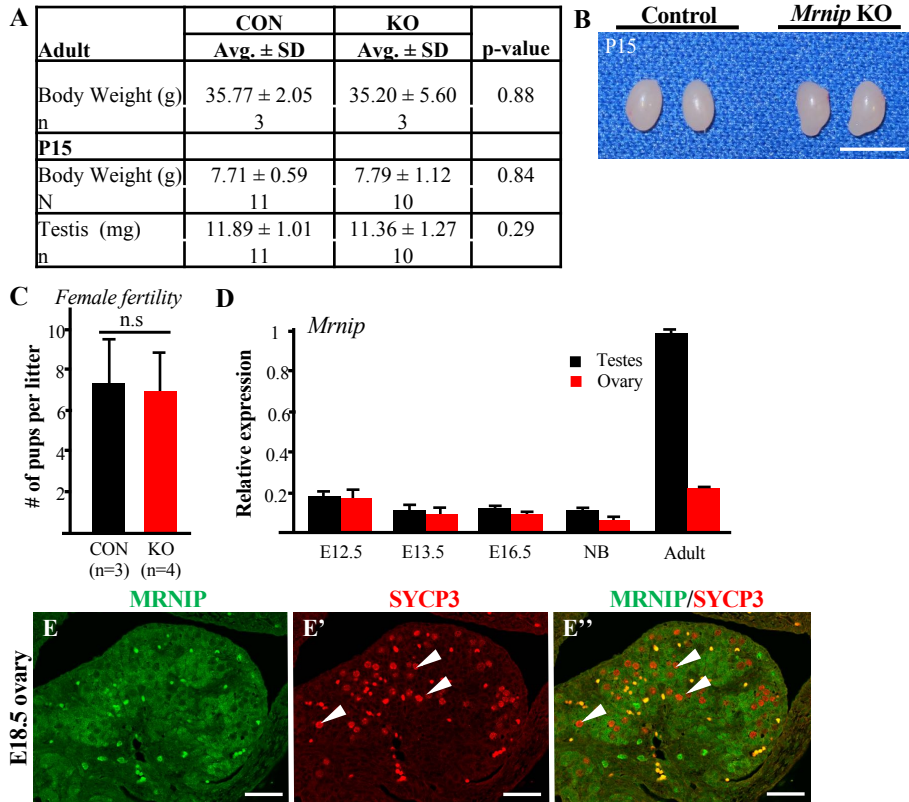

Supplement: Supplementary file 2 — Figure S2 [file FSB2-36-0-s009.pdf]

Fig. S3

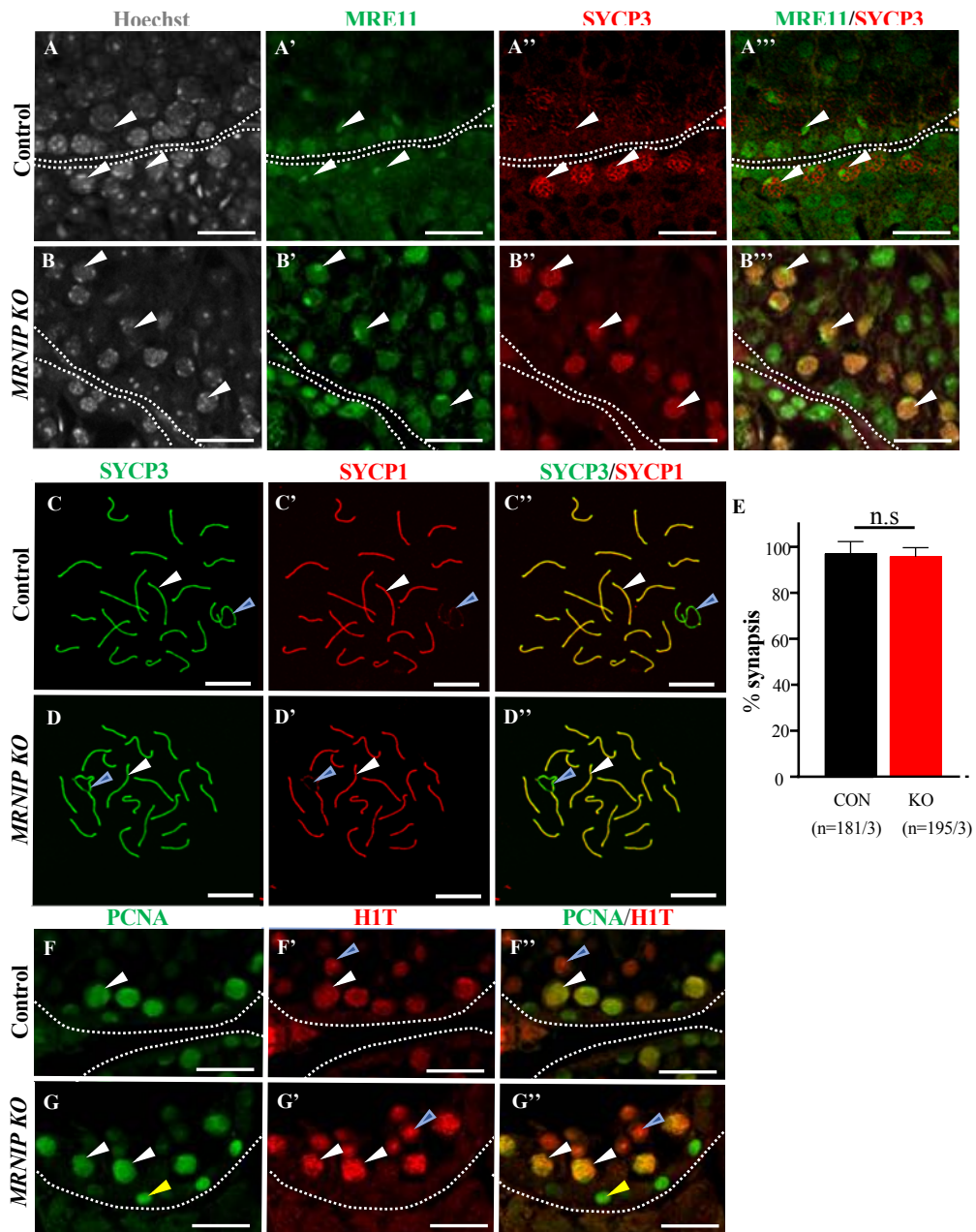

Supplement: Supplementary file 3 — Figure S3 [file FSB2-36-0-s005.pdf]

Fig. S4

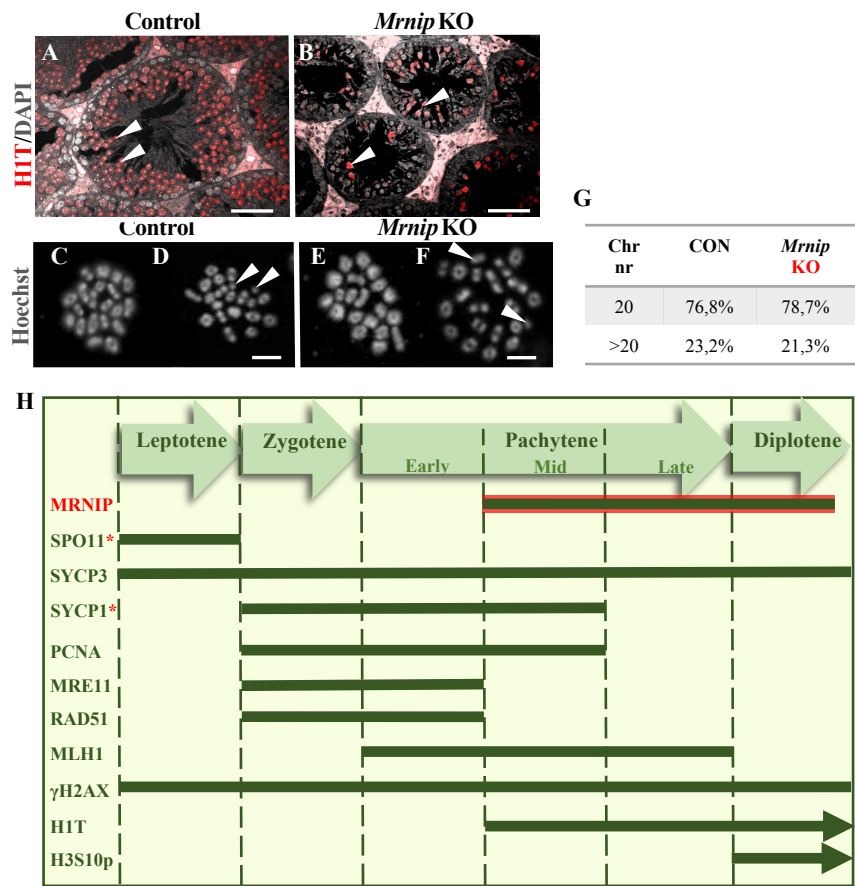

Supplement: Supplementary file 4 — Figure S4 [file FSB2-36-0-s002.pdf]
