# Supplementary material for: MRNIP interacts with sex body chromatin to support meiotic progression, spermatogenesis, and male fertility in mice
Source: FASEB J. 2022 Aug 3;36(9):e22479. doi: 10.1096/fj.202101168RR (PMC9544956; doi:10.1096/fj.202101168RR)
Supplement: Supplementary file 5 — Table S1 [file FSB2-36-0-s007.pdf]

Gene	Forward	Reverse	Reference
**Genotyping**			
WT	TCGTTGGGTTTGTTTGGTTG	CATGTCTTTCTGACCATCCCAC	
Mrnip KO	TCGTTGGGTTTGTTTGGTTG	TCGTGGTATCGTTATGCGCC	
**RT-PCR**			
Mrnip	TCAGGCGCATCAGGTGAAGAAG	CTCCACAGACCTGAGTGACAGC	
Mrnip	GAGAAGCAGTCATTTGTTCGG	ATTTCCTCTTCCTGGGTTGG	
Hprt	TGGATATGCCCTTGACTATAATGAG	TGGCAACATCAACAGGACTC	
hMRNIP	CCAAGAACTGGAGCTGGAAG	CTGCTGCCTTGTTTCACCTT	
hGAPDH	AATCCCATCACCATCTTCCAG	ATGACCCTTTTGGCTCCC	
**qRT-PCR**			
Fthl1	CAGCAGGTCGACATTTTGAA	GGCTGAGCTTGTCAAAGAGG	31
Usp26	GAGGCCCAAAAGTACCAACA	TTCCTGGGAGATTGGTTTTG	31
Rbmy	CAAGAAGAGACCACCATCCT	CTCCCAGAAGAACTCACATT	31
Ube1y1	TTCTTCCAAAAGCTGGATGG	TTCCAGCAGAGGCTTACGAT	31
Mlh1	GGGAGGACTCTGATGTGGAA	ACTCAAGACGCTGGTGAGGT	31
Tex11	TATCAGATTCCCTGGAACTGG	GCACCCTCAAAACAAGCTATG	63
Rbmx	AGAGACGAATGAGAAAGCCCT	AGTGACAAAAGCGAATCCTCTTG	PB:6755296a1
Pgk1	ATGTCGCTTTCCAACAAGCTG	GCTCCATTGTCCAAGCAGAAT	PB:6679291a1
Pgk2	GTTGTAAAGGCCACCTCCAA	ACATACGGGCGTCTTGATTC	20
Ddx3y	GGGTCTGTGATAAGGACAGTTCA	CACGACCACCAATACCATCATAG	PB:25141235a1
Ddx3x	CAGAGTGGAGGAAGTACAGCA	TCACCCCGTGATCCAAAACTG	PB:6753620a1
Utp14a	TGGATTTGACAAGCAACTACCC	CAGACACTTTTAGACCAGCCTC	PB: 12849273a1
Utp14b	AACCCTGATGCCTCTGGAG	TAACTGCTGTATTGTGGGCAC	PB: 26337947a1

Supporting Information Table S1. List of genotyping, RT-PCR, and qRT-PCR primers. PB- Primer Bank.
