# Supplementary material for: MRNIP interacts with sex body chromatin to support meiotic progression, spermatogenesis, and male fertility in mice
Source: FASEB J. 2022 Aug 3;36(9):e22479. doi: 10.1096/fj.202101168RR (PMC9544956; doi:10.1096/fj.202101168RR)
Supplement: Supplementary file 6 — Table S2 [file FSB2-36-0-s003.pdf]

Antibody	Company	Species	Catalog nr	IF dilution	WB dilution
anti-γH2AX	Millipore	mouse	05-636	1:1000	-
anti-SYCP3	Abcam	rabbit	ab15093	1:500	-
anti-SYCP3	Abcam	mouse	ab97672	1:500	-
SYCP1	Abcam	rabbit	ab15090	1:500	-
MRNIP	custom	rabbit	-	1:100	1:1000
ATM	Abcam	mouse	ab78, 2C1	-	1:1000
KAP1	Bethyl Laboratories	rabbit	A300-274A	1:100	1:1000
pKAP1	Bethyl Laboratories	rabbit	A300-767	-	1:1000
MRE11	Novus Biologica	rabbit	NB100-142	1:100	1:1000
RAD50	GeneTex	mouse	GTX70228	-	1:1000
RAD51	Merck	rabbit	PC130	1:100	1:1000
H1T	M. Handel lab	guinea pig	-	1:500	-
H3S10p	Millipore	rabbit	06570	1:200	-
H3S10p	Sigma Aldrich	mouse	05806	1:100	-
B-ACTIN	Sigma Aldrich	mouse	A1978	-	1:5000
B-TUBULIN	Abcam	rabbit	Ab21058	-	1:5000
GAPDH	Millipore	mouse	MAB 374	-	1:5000
B-TUBULIN	Sigma	mouse	T-4026	1:100	-
PCNA	Santa Cruz	mouse	Sc-56	1:100	1:1000
RNA POLII	Millipore	mouse	05623	1:2000	-
MLH1	BD Biosciences	mouse	550838	1:100	
**Secondary antibodies**					
Alexa Fluor anti-rabbit 488	Invitrogen	rabbit	A11008	1:1000	
Alexa Fluor anti-mouse 546	Invitrogen	mouse	A11003	1:1000	
Alexa Fluor anti-rabbit 546	Invitrogen	rabbit	A11010	1:1000	
Alexa Fluor anti-mouse 488	Invitrogen	mouse	A11001	1:1000	
Alexa Fluor anti-guinea pig 594	Invitrogen	guinea pig	A11076	1:1000	
HRP conjugated rabbit anti-mouse Ig	Dako	mouse	P0260		1:5000
HRP conjugated goat anti-rabbit Ig	Dako	rabbit	P0448		1:5000

Table S2. Primary and secondary antibodies, their origin, and dilutions were used in the study.
