# Supplementary material for: MRNIP interacts with sex body chromatin to support meiotic progression, spermatogenesis, and male fertility in mice
Source: FASEB J. 2022 Aug 3;36(9):e22479. doi: 10.1096/fj.202101168RR (PMC9544956; doi:10.1096/fj.202101168RR)
Supplement: Supplementary file 9 — Text S1 [file FSB2-36-0-s001.pdf]

Kazi *et al.* Supplementary Figure Legends

Supporting Information Figure S1. (**A-E''**) MRNIP expression and localization in developing murine testes. (**A-A''**) MRNIP and SYCP3 positive cells are not detected at age P0. (**B-B''**) P5 testes show MRNIP (**B**, green, blue arrows) positive spermatogonia cells. (**C-C''**) P12 testes depicting MRNIP (**C**, green, blue arrows) positive spermatogonia cells and SYCP3 expression in early meiotic cells (**C'**, red, white arrows). Note: MRNIP and SYCP3 do not colocalize in the same cells at P12. (**D-D''**) P15 testes illustrating MRNIP expression in spermatogonia cells (**D**, green, blue arrows) and in pachytene stage meiocytes (**D**, green, white arrows), and SYCP3 (**D'**, red, white arrows) positive spermatocytes. Note: MRNIP and SYCP3 colocalize in mid-pachytene through diplotene stage meiocytes (**D''**, white arrows). Note: occasional MRNIP staining in spermatogonia is detected at P15. (**E-E''**) Adult testes indicating MRNIP (**E**, green, white arrows) and SYCP3 (**E'**, red, white arrows point to double-labeled meiocytes). MRNIP and SYCP3 colocalize from the mid-pachytene stage (**E''**, white arrows). Note: MRNIP staining becomes specific to meiocytes and are not seen in spermatogonia in adult testes. Scale bar (**A-E''**) 50 µm.

Supporting Information Figure S2. (**A**) Average adult weight, and P15 body and testis weight in control and *Mrnip*$^{-/-}$ mice. (**B**) Testes of control (left) and *Mrnip*$^{-/-}$ (right) mice at P15. (**C**) Average number of pups born to control and *Mrnip*$^{-/-}$ females were comparable. (**D**) Relative expression of *Mrnip* in embryonic (E12.5, E13.5, E16.5 and NB) and adult testes and ovaries at different time points, normalized against *Gapdh* and compared to *Mrnip* expression levels in adult testes. (**E-E''**) MRNIP (green) and SYCP3 (red) immunohistochemistry of embryonic ovaries (E18.5). SYCP3 staining confirms that ovaries have meiocytes, but they do not express MRNIP (**E', E'',** white arrows). Student's t-test, ±SD. Scale bar (**B**) 0.5 cm, (**E'-E'''**) 50 µm.

Supporting Information Figure S3. (**A-A'''**) Hoechst (**A**), MRE11 (**A'**, green), SYCP3 (**A''**, red) and merged (**A'''**) antibody staining micrographs of control testis. (**B-B'''**) Hoechst (**B**), MRE11 (**B'**, green) SYCP3 (**B''**, red) and merged (**B'''**) antibody staining micrographs of *Mrnip*$^{-/-}$ testis. White arrows point to spermatocytes with MRE11 expression in sex bodies in control (**A-A'''**) and *Mrnip*$^{-/-}$ (**B-B'''**). (**C-D''**) Nuclear spreads of control (**C-C''**) and *Mrnip*$^{-/-}$ (**D-D''**) labelled with SYCP3 (**C, D,** green) and SYCP1 (**C'**, **D',** red) antibodies showing synapsed (**C-D'',** white arrows synapsis) and unsynapsed sex chromosomes (**C-D''**, blue arrows). (**E**) Quantification of synapsed versus unsynapsed chromosomes did not show difference in control and *Mrnip*$^{-/-}$ meiocytes (CON: n=157/3; KO: n=195/3). (**F-G''**) Immunostaining with PCNA (green) and H1T (red) antibodies in control (**F-F''**) and *Mrnip*$^{-/-}$ (**G-G''**). PCNA and H1T colocalize in mid-pachytene to diplotene stage

spermatocytes in control (**F-F''**, white arrows) and *Mrnip*$^{-/-}$ (**G-G''**, white arrows), but not in spermatogonia (yellow arrows) and spermatids (blue arrows), confirming PCNA expression presence in mid-pachytene to diplotene stage cells in *Mrnip*$^{-/-}$ (**G-G''**). Scale bar (**A-B'''**, **F-G''**) 20 µm and (**C-D''**) 10 µm.

Supporting Information Figure S4. (**A-B**) Immunolabeling of testis cross-section depicts abundant H1T expression in meiocytes and round spermatids in control (**A**, white arrows**)** and a clearly reduced H1T positive cell population in *Mrnip*$^{-/-}$ (**B,** white arrows**).** **(C-F)** Metaphase spreads of paired (**C**, **E**) and unpaired (**D**, **F**, white arrows point to unpaired chromosomes) chromosomes in control (**C**, **D**) and *Mrnip*$^{-/-}$ spermatocytes (**E**, **F**). (**G**) Quantification of paired and unpaired chromosomes illustrating no significant difference between control and *Mrnip*$^{-/-}$ spermatocytes. **(H)** Schematic representation of MRNIP expression in relation to some key meiotic factors presented in this study. Scale bars **(A-B)** 50 µm, **(C-F)** 10µm

Supporting Information Movie S1. Epididymal extract recorded by CASA indicate motile spermatozoa in control *Mrnip*$^{+/-}$ mice. Movie is shown in slow motion (AVI).

Supporting Information Movie S2. Epididymal extract recorded by CASA indicate no motile spermatozoa in *Mrnip*$^{-/-}$ mice. Movie is shown in slow motion (AVI).

Supporting Information Table S1. List of genotyping, RT-PCR, and qRT-PCR primers. PB-Primer Bank.

Supporting Information Table S2. List of primary and secondary antibodies used in the study, their manufacturers, and dilutions.
